# Electrospun Nickel Manganite (NiMn_2_O_4_) Nanocrystalline Fibers for Humidity and Temperature Sensing

**DOI:** 10.3390/s21134357

**Published:** 2021-06-25

**Authors:** Milena P. Dojcinovic, Zorka Z. Vasiljevic, Jugoslav B. Krstic, Jelena D. Vujancevic, Smilja Markovic, Nenad B. Tadic, Maria Vesna Nikolic

**Affiliations:** 1 Institute for Multidisciplinary Research, University of Belgrade, 11030 Belgrade, Serbia; zorkav@imsi.rs (Z.Z.V.); mariavesna@imsi.rs (M.V.N.); 2 Institute of Chemistry, Department of Catalysis and Chemical Engineering, Technology and Metallurgy, University of Belgrade, 11000 Belgrade, Serbia; jkrstic@nanosys.ihtm.bg.ac.rs; 3 Institute of Technical Sciences of SASA, 11000 Belgrade, Serbia; jelena.vujancevic@itn.sanu.ac.rs (J.D.V.); smilja.markovic@itn.sanu.ac.rs (S.M.); 4 Faculty of Physics, University of Belgrade, 11000 Belgrade, Serbia; nenad.tadic@ff.bg.ac.rs

**Keywords:** nickel manganite, electrospinning, nanocrystalline fibers, humidity sensing, temperature sensing, NTC thermistor

## Abstract

Nickel manganite nanocrystalline fibers were obtained by electrospinning and subsequent calcination at 400 °C. As-spun fibers were characterized by TG/DTA, Scanning Electron Microscopy and FT-IR spectroscopy analysis. X-ray diffraction and FT-IR spectroscopy analysis confirmed the formation of nickel manganite with a cubic spinel structure, while N_2_ physisorption at 77 K enabled determination of the BET specific surface area as 25.3 m^2^/g and (BJH) mesopore volume as 21.5 m^2^/g. The material constant (B) of the nanocrystalline nickel manganite fibers applied by drop-casting on test interdigitated electrodes on alumina substrate, dried at room temperature, was determined as 4379 K in the 20–50 °C temperature range and a temperature sensitivity of −4.95%/K at room temperature (25 °C). The change of impedance with relative humidity was monitored at 25 and 50 °C for a relative humidity (RH) change of 40 to 90% in the 42 Hzπ1 MHz frequency range. At 100 Hz and 25 °C, the sensitivity of 327.36 ± 80.12 kΩ/%RH was determined, showing that nickel manganite obtained by electrospinning has potential as a multifunctional material for combined humidity and temperature sensing.

## 1. Introduction

Monitoring humidity and moisture levels is essential in many industrial processes, such as the food, medical and semiconductor industries, as high-quality products often need to be produced in controlled conditions [1]. Humidity sensors are components of smart monitoring systems [2]. In the food industry, besides humidity monitoring in the food production process, humidity sensors can be incorporated as components of intelligent food packaging [3]. Precision agriculture requires smart humidity sensors as part of a wireless sensor network [4,5], that includes monitoring of plant soil moisture essential for good plant growth [6,7]. Semiconductor materials, especially metal oxides, are widely applied in gas and humidity sensors [8] and remain the focus of much research [9,10]. A good sensing material needs to show high sensitivity, good selectivity, and be low-cost, reliable and easy to fabricate [8]. The rapid development of flexible electronics increases the need for developing sensing materials suited for such applications [11,12], such as metal oxide capacitive humidity sensors on paper [2]. Recent research has focused on developing a self-powered, flexible humidity sensor [13]. Miniaturized flexible temperature monitoring using negative temperature coefficient (NTC) materials, such as NiO, has also been a subject of research [14]. In human health monitoring, besides temperature [15], respiration monitoring can be performed by impedance-type humidity sensors [16]. A combined multifunctional spinel ceramic humidity and temperature sensing potential was investigated by Vakiv et al. [17].

Nickel manganite is a well-known NTC thermistor oxide material, low-cost, non-toxic and abundant [18], with a cubic spinel structure and a small polaron hopping conduction mechanism between manganese cations (Mn^3+^ and Mn^4+^) [19,20]. It has extensively been investigated as an NTC thermistor in bulk, thick or thin film form [19,21,22,23]. Recently, nickel manganite has been the subject of much research for energy storage as a supercapacitor, [24,25] in photocatalysis [26], or as anode material for lithium-ion batteries [27]. Gawli et al. investigated the possible application of NiMn_2_O_4_ as a humidity sensing material [28]. The structure and morphology of NiMn_2_O_4_ as a result of the synthesis method have a noticeable influence on electrical properties [23]. Different methods have been used to synthesize NiMn_2_O_4_ nanoparticles, such as sol–gel [25], solution combustion [20,27,29] and hydrothermal methods, [30] resulting in different particle morphology and properties. High surface area, mesoporous and aligned NiMn_2_O_4_ nanofibers were obtained by Bhagwan et al. [31] using electrospinning that showed improved energy storage properties.

Mesoporous metal oxides are widely applied as gas sensing materials, including humidity sensing [32]. A large surface area provides more active sites for adsorption of water molecules on the surface of the sensing material, while the porosity, derived from the right system of pores, allows better exposure to available active sites, facilitates the adsorption of H_2_O in the response phase of sensor use and does not hinder the desorption of H_2_O molecules in the recovery phase [33]. According to Wang et al. [34], an increase in reaction sites due to a high specific surface area of the sensing material can enhance the gas sensor activity and sensitivity. A highly porous structure with a well-connected system of transport channels suitable for fast diffusion of gas molecules results in highly sensitive sensors with a fast response and recovery [32]. Optimization of the pore structure in gas sensing materials can also result in improved selectivity [34].

In this work, we used electrospinning to obtain NiMn_2_O_4_ nanocrystalline fibers with a high specific surface area and mesoporous structure with potential as a multifunctional material, investigated here for humidity and temperature sensing.

## 2. Materials and Methods

### 2.1. Materials

Nickel nitrate hexahydrate (Ni(NO_3_)_2_⋅6H_2_O, purum p.a., purity ≥ 97%), manganese acetate tetrahydrate ((CH_3_COO)_2_Mn⋅4H_2_O, ACS reagent, purity ≥ 99%), N,N-Dimethylformamide (DMF, puriss, ACS reagent, ≥99.8%), absolute ethanol (ACS reagent), polyvinylpyrrolidone (PVP, M*_w_* = 1,300,000) and deionized water were purchased from Merck (Sigma Aldrich, Darmstadt, Germany) and used as received.

### 2.2. Electrospinning

**Figure 1:**
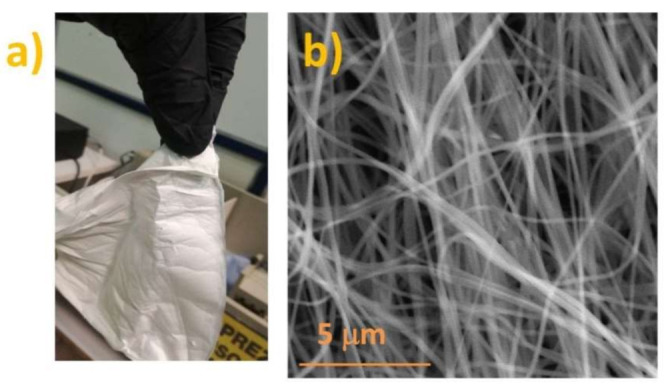
Photo (**a**) and SEM image (**b**) of as-spun fiber peeled off the baking paper.

Nickel nitrate hexahydrate and manganese acetate tetrahydrate in the molar ratio of 1:2 were dissolved in distilled water and mixed with 5 mL of DMF dissolved in 5 mL of ethanol. The solution was mixed on a magnetic stirrer until all components were completely dissolved. PVP was slowly added to make a 10% polymer solution that was left to mix overnight. The mixture was loaded into a plastic syringe with a 0.8 mm diameter needle connected to a high voltage supply in an “in-house” designed electrospinning system. The applied voltage was maintained at 21.5 kV, while the rotating drum collector speed was 1200 rpm. The distance between the tip of the needle and the rotating drum collector was set at 12 cm, while the solution was pumped from the syringe at 1.5 mL/h. The fibers were collected on baking paper mounted on the rotating drum collector. As-spun fibers (as shown in Figure 1a) were peeled off from the paper and calcined in a chamber furnace in air at 400 °C for 4 h with a slow heating rate of 1°/min, in accordance with the literature [31].

### 2.3. Characterization of As-Spun Fibers

Simultaneous TG/DTA analysis of as-spun fibers was performed on a SETSYS Evolution 24,000 Setaram Instrumentation device (Cailure, France) up to 1000 °C with a heating rate of 5°/min in air. FT-IR spectroscopy of the as-spun fibers was performed on a FT-IR Nicolet 6700 ATR device (Thermo Fisher, UK) with the resolution of 4 cm^−1^, in the 400–4000 cm^−1^ range. The as-spun fiber morphology was observed by scanning electron microscopy (SEM) on a TESCAN Electron Microscope VEGA TS 5130MM (Czech Republic).

### 2.4. Characterization of NiMn_2_O_4_ Nanocrystalline Fibers

The crystal structure of NiMn_2_O_4_ nanofibers was analyzed using X-ray diffraction (Rigaku Ultima IV diffractometer, Japan) in the 10–90° range. Functional groups were determined by FT-IR spectroscopy (on the same instrument and conditions as above). The fiber morphology was observed by field emission scanning electron microscopy (FESEM) on a TESCAN MIRA3 XM (Czech Republic). The nitrogen adsorption–desorption isotherm was collected on a Sorptomatic 1990 Thermo Finigen (Thermo Fisher, UK) at 77 K. Samples for measurement were prepared by degassing in a vacuum at 110 °C for 36 h.

### 2.5. Humidity and Temperature Sensing

NiMn_2_O_4_ nanocrystalline fiber powder was ground for a short time with an agate mortar and pestle, mixed with a small amount of deionized water to prepare a paste that was drop cast onto alumina substrate with small test interdigitated PdAg electrodes (finger width 0.6 mm, finger spacing 0.3 mm), previously prepared by screen printing PdAg paste onto alumina substrate combined with firing in a conveyor furnace at 850 °C for 15 min, as shown in detail in [35]. The thick film samples were dried and left to age at room temperature for several days. Change in impedance with relative humidity (RH 40–90%) was measured on a HIOKI 3532-50 LCR HiTESTER in the 42 Hz − 1 MHz frequency range and the set voltage of 1 V in a JEIO TECH TH-KE-025 temperature and humidity climatic chamber. Two operating temperatures were selected, 25 and 50 °C. The desired temperature and humidity values were obtained by initially setting the temperature and starting humidity (RH 90%). Stable temperature and humidity was established for on average 30–45 min in the temperature and humidity climatic chamber. Then, the sample was measured and a new humidity value was set, while the temperature was maintained at a constant value. When the set RH and temperature stabilized, the impedance was measured and another RH value was set. DC resistance was measured on a Tektronix DMM4040 6-1/2 digit precision multimeter in the same temperature and humidity climatic chamber while maintaining constant humidity, with RH set at 40% in the temperature interval 20–50 °C. The desired humidity and temperature values were obtained by setting RH at 40% and the initial temperature at 20 °C. When this was established (after 30–45 min) the resistance was measured, and another temperature value was set, while the RH was constant. When the set temperature value stabilized, the sensor resistance was measured again.

## 3. Results and Discussion

### 3.1. As-Spun Fibers

As-spun fibers were smooth and randomly oriented with a uniform surface (Figure 1). The mean fiber diameter was determined by measurement of the width of 40 nanofibers in recorded SEM images using the Gatan Micrograph^®^ software as 238 nm with values ranging from 130 to 430 nm.

**Figure 2:**
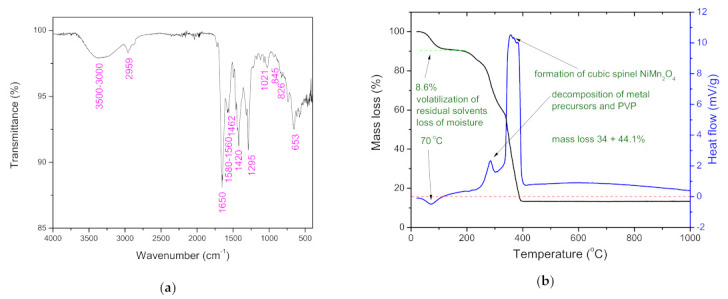
FT-IR spectrum (**a**) and TG/DTA plots (**b**) measured on as-spun fibers.

The FT-IR spectrum of as-spun fiber (Figure 2a) shows bands originating from PVP, DMF, nickel nitrate and manganese acetate precursors. OH group vibrations in the 3000–3500 cm^−1^ range are noted, belonging to residual water in the fibers [36,37,38]. The bending vibration of the OH group, usually noted around 1635 cm^−1^ [38] is possibly present but masked by the C=O stretching vibration noted at ≈1650 cm^−1^ of the carbonyl group in PVP [39]. C−H stretching, and bending vibrations of the CH_2_ group in PVP were noted at ≈2959 cm^−1^ and 1420 cm^−1^, respectively [37,39]. Tertiary amine C−N stretching vibrations can be noted at ≈1290 cm^−1^ [40], while absorption of the C−N vibration of PVP can be noted at ≈1021 cm^−1^ [37]. CH_2_ rocking vibrations of PVP can be noted at ≈845 cm^−1^ [39]. The bands in the range of 1580 to 1560 cm^−1^ and ≈1462 cm^−1^ can be assigned to asymmetric and symmetric vibrations of C−O originating from the acetate ion in the manganese acetate precursor [41]. Vibrations of nitrate groups in the nickel nitrate precursor expected at 1620 and 1380 cm^−1^ are masked by the C=O stretching vibration and C−H bending vibrations of PVP, respectively [42]. A small vibration band at 826 cm^−1^ can be attributed to the bending mode of the nitrate group [37], while the vibration band at 653 cm^−1^ can be due to both the vibrational mode of nitrate groups in the nickel nitrate precursor, and Mn−O vibration in the manganese acetate precursor [37,41].

The measured TG/DTA in Figure 2b shows the thermal behavior of the as-spun fibers. The total mass loss was ≈86.7% up to 400 °C, after which it remained constant in the temperature region 400–1000 °C. The mass loss can be divided into three regions in accordance with Bhagwan et al. [31] who determined the same for electrospun nickel manganite fibers. It started with volatilization of the residual solvents present in fibers (DMF, ethanol) and loss of moisture/desorption of water (H_2_O) [36] corresponding to the initial minor mass loss of ≈8.6% from ≈20 to 113 °C, and endothermic peak at ≈70 °C. A small exothermic peak at ≈284 °C, followed by a distinct wide exothermic peak with a maximum at ≈357 °C can be noted, accompanied by a weight loss of 34 and 44.1%, respectively. Both peaks may be ascribed to decomposition of the metal precursors and PVP in the electrospun fibers, but the second can be ascribed to complete decomposition and removal of PVP and formation of the cubic spinel structure [31,37].

### 3.2. NiMn_2_O_4_ Nanofibers

**Figure 3:**
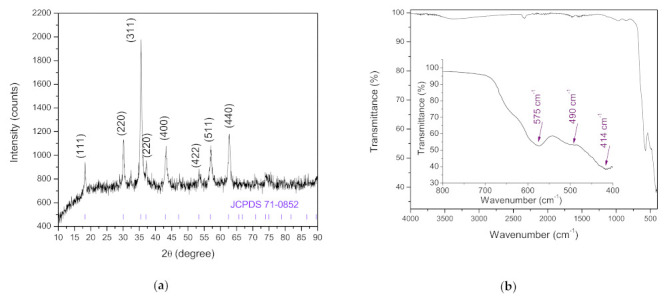
XRD diffractogram (**a**) and FT-IR spectrum (**b**) measured on NiMn_2_O_4_ nanocrystalline fibers.

Calcination of the as-spun fibers resulted in the formation of NiMn_2_O_4_ nanocrystalline fibers. The characteristic reflections on the measured X-ray diffraction pattern (Figure 3a) could be assigned to the $Fd\bar{3}m$ cubic spinel structure, in accordance with the ICCD/JCPDS card 71-0852 [43]. The measured FT-IR spectrum (Figure 3b) showed that all organics from PVP have burnt out, leaving only a small incline at ≈3385 cm^−1^ and ≈1641 cm^−1^ from OH group stretching and bending bands due to readsorbed atmospheric H_2_O in the NiMn_2_O_4_ spun fibers. This was noted before for electrospun NiFe_2_O_4_ fibers [37]. The noticeable bands at ≈575, 414 and the small incline at ≈490 cm^−1^ can be attributed to vibration bands originating from tetrahedral and octahedral groups of Mn^3+^−O, Ni^2+^−O [43,44] and, according to Bhagwan et al. [31], also Ni−O−Mn vibrations in NiMn_2_O_4_, confirming the formation of NiMn_2_O_4_ after calcination.

**Figure 4:**
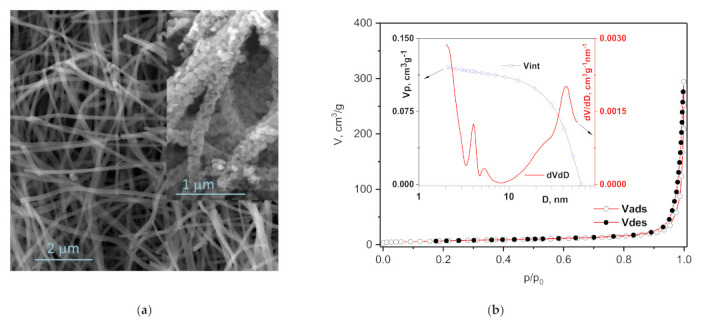
FESEM images (**a**) and nitrogen adsorption–desorption isotherms (**b**) of NiMn_2_O_4_ nanocrystalline fibers inset: pore size distribution.

The morphology of NiMn_2_O_4_ nanofibers is shown in Figure 4a. The fiber network structure remained after calcination. The fiber width decreased due to removal of the polymer component (PVP backbone) and crystallization of the metal component, as shown before in the literature [31,45,46,47]. The average fiber diameter was 207 nm, with values ranging from 160 to 250 nm. The fibers are composed of small interconnected NiMn_2_O_4_ nanoparticles (as shown in the inset in Figure 4a) in the range of 20 to 50 nm, similar to the morphology obtained by Bhagwan et al. [31].

The measured nitrogen adsorption–desorption isotherm (Figure 4b) is of type II, according to the IUPAC nomenclature [48]. However, the obvious presence of hysteresis of the loop, in the region of relative pressure (p/p_0_) higher than 0.8, reveals the presence of high diameter mesopores, as well. The pore size distribution curve is given as an inset in Figure 4b and shows pores ranging from 2 to 63 nm including macropores > 50 nm besides mesopores (2–50 nm). Indeed, the value of mesopore volume (0.089 cm^3^/g), determined by the BJH method, represents 52% of the total pore volume determined by the Gurevich method for p/p_0_ = 0.99. Additionally, the most significant contribution to the total specific surface area of 25.3 m^2^/g, calculated using the Brunauer–Emmett–Teller (BET) equation, originates from the mesopores’ specific surface area of 21.5 m^2^/g (calculated with the BJH method). In contrast, the volume of micropores determined by the t-plot method is small; 0.005 cm^3^/g. Overall, similar to Bhagwan et al. [31], we obtained high surface area mesoporous NiMn_2_O_4_ nanocrystalline fibers.

### 3.3. NTC Temperature Dependence of DC Resistance

**Figure 5:**
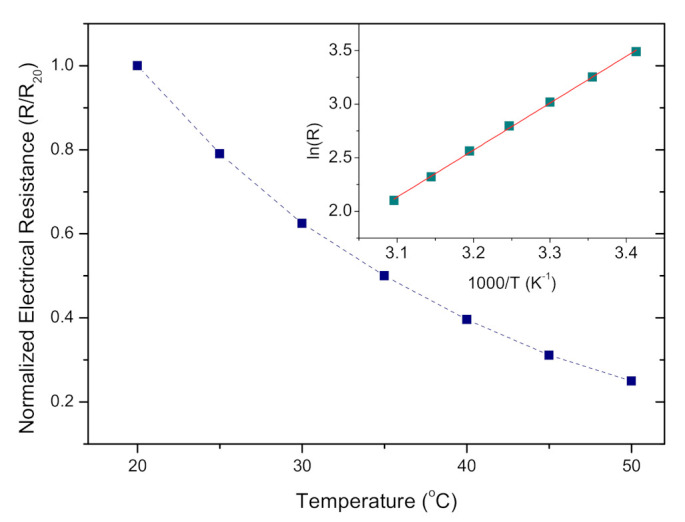
Electrical resistance of NiMn_2_O_4_ nanocrystalline fibers; inset represents an estimation of the material constant (B).

As noted before for NiMn_2_O_4_ [18,19,29] as an NTC thermistor material, the measured DC resistance decreased with an increase in temperature (20–50 °C), following an exponential Arrhenius dependence [18], as shown in Figure 5 and the equation

(1) $$R_{T}=R_{\infty }e^{B/T}$$

where *R*_∞_ is the reference resistance of the thermistor, representing resistance at an infinite temperature when 1/*T* = 0, *T* is the temperature expressed in K, and B is the material constant (or B-value) of the thermistor.

In our case, the change in resistance was monitored in a temperature and humidity climatic chamber, maintaining a constant relative humidity (RH) of 40% (the most common value of RH in our laboratory and surrounding environment) and changing the temperature, as the change in RH had an influence on overall resistance values.

The material constant (B) of the thermistor (B-value) was estimated from the linear fit (as shown in the inset in Figure 5) as 4397 K in the temperature interval 20–50 °C. This value is within the range used in commercial NTC bulk ceramics (2000–5000 K) [18,49], slightly lower than the value of 4812 K determined for NiMn_2_O_4_ powder obtained by sol–gel combustion [29], lower than for LaNiO_3_-NiMn_2_O_4_ nanocomposite thick films [50] where B was over 5000 K, or the significantly high B value of 7350 K obtained for NiO temperature sensors by Shin et al. [15], all obtained at room temperature. Wang et al. obtained the B-value of 4310 K for miniature nickel oxide thick film thermistors printed using aerosol jet technology [51]. Schubert et al. [52] obtained the B-value of 4250 for aerosol as-deposited films, that decreased to the 3500–3900 K range with film firing in the temperature interval of 200 to 600 °C. Bulk nickel manganite NTC thermistors most often have B-values in the 3500–3900 K range, as recently determined by Li et al. [53] for a core–shell NTC ceramic, or 3500 K determined by Reiman et al. [54] for a multilayer Ni-Zn-Co-Mn-O NTC ceramic fired at 900 °C.

The temperature sensitivity at room temperature (25 °C) was determined as α = −4.95%/K = 1/*R*⋅d*R*/d*T* = −*B*/*T*^2^. This value is comparable with commercial devices (−4%/K) [18,49] and confirms the potential of application of NiMn_2_O_4_ nanocrystalline fibers in temperature sensing, especially as these values were obtained for drop-cast powder with no further temperature treatment. NiMn_2_O_4_ nanocrystalline fibers could be applied in temperature sensors in the form of ink on flexible substrates that can tolerate only low-temperature treatment of the sensing layer [15].

### 3.4. Influence of Humidity on Complex Impedance

**Figure 6:**
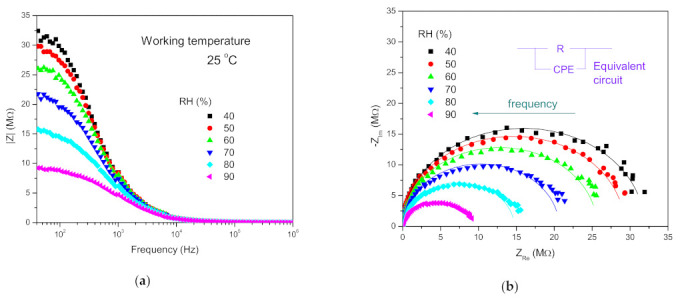
Impedance dependence on frequency (**a**) and complex impedance (**b**) measured (points) and fitted (line) using the equivalent circuit shown in the inset, for relative humidity RH 40-90% of NiMn_2_O_4_ nanocrystalline fibers at the working temperature of 25 °C.

At both operating temperatures (25 and 50 °C), the measured impedance decreased with the increase in relative humidity (RH), and also with the change in frequency, as shown in Figure 6a for the operating temperature of 25 °C. The complex impedance magnitude becomes smaller with an increase in RH, as shown in Figure 6b.

**Figure 7:**
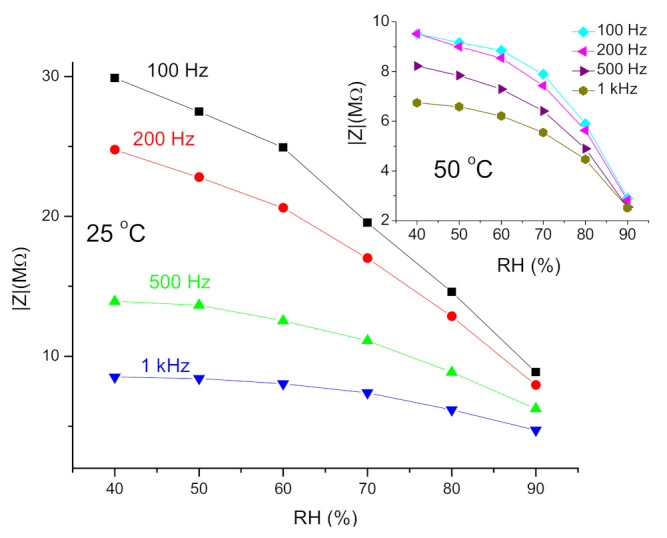
Impedance dependence on RH measured at selected frequencies.

One dielectric relaxation process was noted for complex impedance. This has been noted before for metal oxide semiconductors, including nickel manganite [20,28,55,56]. Highly conducting grains and the resistive nature of grain boundaries present in nickel manganite contribute to this relaxation process [57]. One semicircle shows that either one relaxation process (originating from grains or grain boundaries) is dominant, or grain and grain boundary contributions overlap [58,59]. Adsorption of water molecules by a surface reaction mechanism with the nanocrystalline fiber particles of NiMn_2_O_4_ with a rise in RH leads to an increase in ionic conductivity in accordance with the humidity sensing mechanism [35,60]. This was most noticeable in the lower frequency range, as shown in Figure 7 for several selected frequencies.

The sensitivity, defined as the ratio between the change of sensor impedance and RH (ΔZ/ΔRH) at 100 Hz, was determined as 327.36 ± 80.12 kΩ/%RH at 25 °C. It decreased with an increase in frequency, and was 259.33 kΩ/%RH, 93.34 kΩ/%RH, 40.71 kΩ/RH at 200, 500 and 1000 Hz, respectively. At the working temperature of 50 °C, the sensitivity at 100 Hz was determined as 69.88 kΩ/%RH. A similar decrease in sensitivity with frequency was noted by Zhang et al. [60]. The obtained sensitivity value at 100 Hz of 327.36 kΩ/%RH is comparable with sensitivity data determined for NiO/SnO_2_ electrospun fibers [61] and nanocrystalline Zn_2_SnO_4_/SnO_2_ composite [35], confirming the potential of NiMn_2_O_4_ nanocrystalline fibers for application in humidity sensing.

The measured complex impedance spectra (as shown in Figure 6b at the working temperature of 25 °C) were analyzed using an equivalent circuit consisting of a parallel resistance and constant phase element (CPE), as it was not possible to separate the influence of grain and grain boundary components of the measured impedance, as shown in the inset in Figure 6b. The CPE element was used, as we expected non-ideal Debye capacitance behavior previously noted for NiMn_2_O_4_ synthesized by refluxing nickel oleate and manganese oleate [28], and other metal oxides, such as FeMnO_3_ [55] or Fe_2_TiO_5_ [56]. Fitting of experimental data to the proposed equivalent circuit was performed using the EIS Spectrum Analyzer Software [62]. Good agreement was obtained between measured and fitted data, as shown in Figure 6b. The small deviation at low frequencies for high humidity can be attributed to a combined grain boundary and electrode effect. The obtained values determined for resistance, capacitance and the critical exponent (*n*) are shown in Table 1.

The capacitance was calculated taking into account the case of parallel resistance and CPE element, as described in detail in [58]. The determined capacitance increased slightly with an increase in RH in a similar range for both working temperatures. Its value was between 19.8 and 29.1 pF, indicating the dominant influence of grains, as grain capacitance values fall in this range, as noted previously for NiMn_2_O_4_ powder obtained by sol-gel auto-combustion [20], or Ni_0.54_Mn_1.26_Fe_1.2_O_4_ NTC ceramic thermistors obtained by spark plasma sintering [59]. In the case of electrospun NiMn_2_O_4_ nanocrystalline fibers, at low relative humidity values (RH 40, 50%) at both working temperatures, the dependence of complex impedance on frequency represented an ideal semicircle (the critical exponent *n* was one). As the relative humidity increased the *n* value decreased, though it remained relatively close to 1, reaching 0.96038 and 0.95068 for RH 90% at the working temperatures of 25 and 50 °C, respectively. This shows that the behavior of NiMn_2_O_4_ nanocrystalline fibers was close to perfect Debye, with the measured complex impedance semicircle showing only a slight depression that increased with an increase in RH from 60 to 90%. Similar values of the critical exponent (between 0.97 and 1) were noted before for NiMn_2_O_4_ thin films, but for the change in temperature in the 180–500 K range [63].

**Figure 8:**
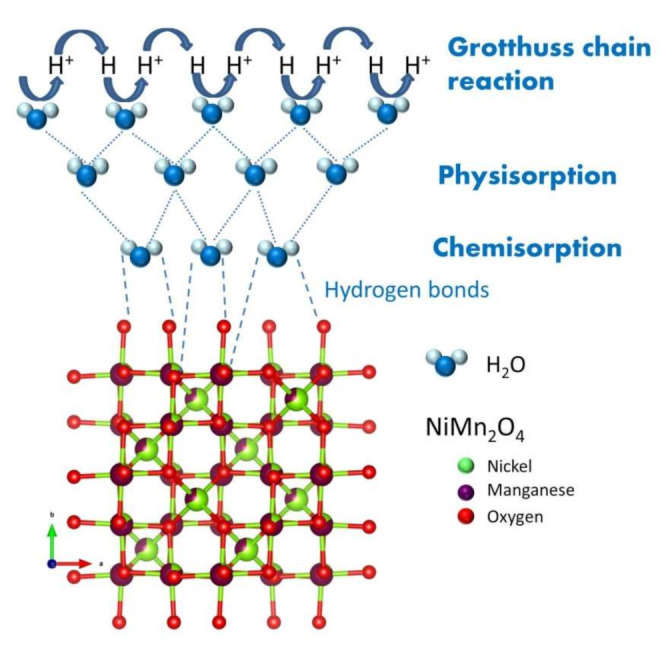
Humidity sensing mechanism of nickel manganite nanocrystalline fibers, NiMn_2_O_4_ $Fd\bar{3}m$ cubic spinel structure drawn using VESTA [64].

The dielectric relaxation frequency was determined as 2π*f_rel_* = 1/*R⋅C* and it shifted towards higher values with an increase in RH, and also with working temperature (as shown in Table 1). The determined resistance decreased noticeably with the increase in RH similar to the impedance decrease, reflecting the water adsorption (humidity sensing) mechanism (shown in Figure 8) by NiMn_2_O_4_ nanocrystalline fibers.

At low RH, water molecules are chemisorbed by the mesoporous nanocrystalline NiMn_2_O_4_ fibers on the sample surface. Hydrogen bonds form firmly attached OH groups. With the increase in RH, more water molecules are physically adsorbed on the chemisorbed layer. High RH leads to the formation of multiple physisorbed layers. Mobility of water molecules is enabled by single hydrogen bonding with H^+^ acting as the dominant charge carrier. A Grotthuss chain reaction (charge transport mechanism) is used to describe the conductivity [65]. A proton is released by H_3_O^+^ to a water molecule that is close by and ionized, forming H_3_O^+^ again. This results in proton hopping between water molecules, increased conductivity, reduced resistance, shrinking the complex impedance semicircles and a shift in the dielectric relaxation frequency towards higher values.

## 4. Conclusions

High surface (25.3 m^2^/g) mesoporous nanocrystalline nickel manganite fibers with a cubic spinel structure were obtained using the electrospinning method, followed by calcination at 400 °C with a slow heating rate of 1°/min. Drop-casting a paste made from the obtained powder and water on test interdigitated alumina electrodes enabled determination of the B constant as 4379 K in the 20–50 °C temperature range, and a temperature sensitivity of −4.95%/K at 25 °C. The sensitivity of 327.36 kΩ/%RH in the relative humidity range of 40 to 90% confirmed the potential of electrospun nanocrystalline NiMn_2_O_4_ fibers as a multifunctional material for combined temperature and humidity sensing.

## Figures and Tables

**Table 1:** Resistance, capacitance and critical exponent calculated from measured complex impedance spectra using the equivalent circuit composed of a parallel resistance and CPE element.

**Working Temperature 25 °C**				
**Relative Humidity (%)**	**R (MΩ)**	**C (pF)**	**n**	**f_rel_ (Hz)**
40	31.898	20.151	1	1555
50	29.272	20.142	1	1696
60	25.693	20.542	0.99807	1894
70	20.644	21.903	0.98948	2211
80	14.750	24.264	0.97599	2794
90	8.823	27.206	0.96038	4262
**Working Temperature 50 °C**				
**Relative Humidity (%)**	**R (MΩ)**	**C (pF)**	**n**	**f_rel_ (Hz)**
40	9.727	19.852	1	5178
50	9.479	19.972	1	5282
60	8.768	20.293	0.99868	5622
70	7.643	21.126	0.99234	6192
80	5.715	22.577	0.98297	7749
90	2.806	29.096	0.95068	12,245

## Data Availability

The data presented in this study are available on request from the corresponding author. The data are not publicly available due to ongoing research.
